# Midwifery as a family health worker in the surveillance system during the COVID-19 pandemic in Turkey

**DOI:** 10.7189/jogh.12.03043

**Published:** 2022-07-06

**Authors:** Hafize O Can, Ferhan Sahin, Elif Nacaroglu

**Affiliations:** 1 Ege University Faculty of Health Sciences, Midwifery Department, Izmir, Turkey; 2 TR Ministry of Health Kütahya Central Community Health Center Kütahya, Turkey; 3 TR Ministry of Health Turgutlu Family Health Center, Manisa, Turkey

The coronavirus disease has spread rapidly due to being highly transmittable between humans. As in the rest of the world, in Turkey, The Ministry of Health (MoH) has published guidelines, algorithms, and flowcharts for fighting against COVID-19. Efforts have been initiated to reduce the rate of spread of the disease in the community by having the primary, secondary, and tertiary health care institutions take quick steps such as case detection, examination, and follow-up of epidemiological sources and contact tracing during the pandemic in Turkey [1].

**Figure:**
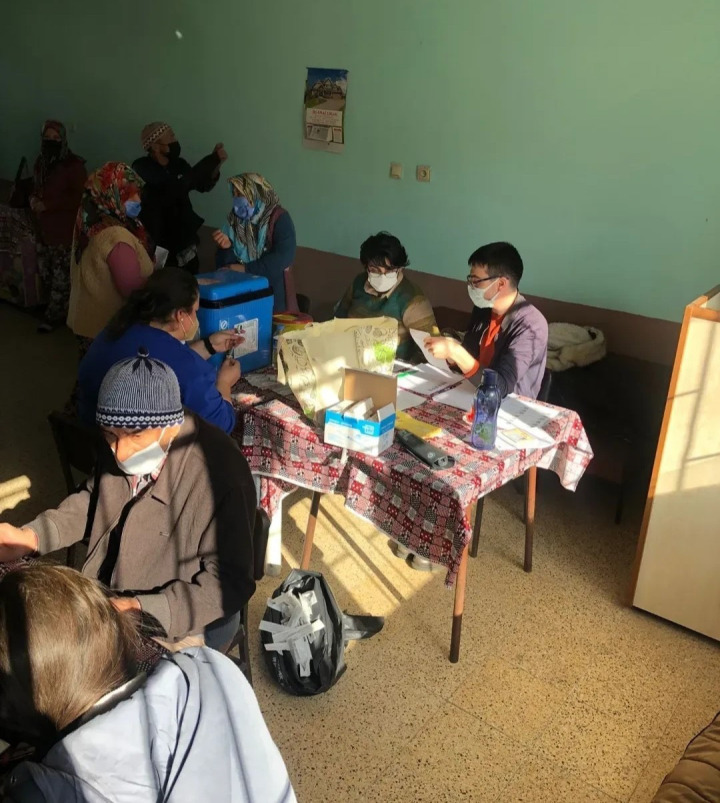
Photo: COVID-19 vaccination studies of Family Health Workers. Source: Elif Nacaroğlu's personal collection, used with permission).

The COVID-19 pandemic has necessitated a transformation from patient-centred to community-centred care. Pandemic solutions were needed not only for hospitals but also for the entire population [2]. COVID-19 has led to the remodelling of the structures and working processes of health services, especially in primary health care [3]. With the pandemic, issues such as case registration and detection, isolation and follow-up of contacts have come to the fore. This process is equally or more important than the effectiveness of the treatment. Within primary care services (which include preventive health services), surveillance efforts are required to rapidly identify and test symptomatic individuals so that community status could be observed. Active surveillance is crucial in taking and developing effective measures for controlling and preventing infection by understanding a disease’s transmission patterns. The basic aim is to isolate and cut off the transmission path by following the chain of transmission [4].

Midwives are one of the many health professionals who have been at the core of the fight against the pandemic [3]. During this time, many women became pregnant and gave birth. These women and their families continued to need midwifery support and care. To achieve the best health outcomes for women and newborns during the pandemic, up-to-date information on COVID-19 and evidence-based studies were used to inform maternity care [5-7].

Postponing births and midwifery support for women’s needs during the pandemic is impossible. Besides the services provided during the pandemic, obstetric and postpartum midwifery care has been added to the follow-up of pregnant women who were in close contact with a person with COVID-19 [8]. Midwives have been the starting point for the planning of necessary interventions such as infectious disease control, early diagnosis of cancer, family planning counselling for the prevention of unwanted pregnancies, pre-marital counselling, and others, with the aim of determining the public health issues, problems, and priorities.

The purpose of this review is to determine the duties and responsibilities of midwives working in Family Health Centers (FHC) in the surveillance system during the COVID-19 pandemic in Turkey.

56 531 midwives are actively working in Turkey. 52 495 of them work in institutions affiliated with the MoH; 20.62% in Community Health Centers (CHC) and Health Houses (primary care service) affiliated with provincial and district health directorates, 59.61% work in hospitals (secondary care hospitals), and 19.77% work in FHC as family health workers (primary care service). Within the scope of primary health care, midwives carry out community-based midwifery services [9]. Community-based health services are shown in **Figure 1**. Midwives working in Family Medicine are called Family Health Workers. Together with family physicians, they are responsible for providing preventive, therapeutic, and rehabilitative health services for the person in a team approach and keeping health records and statistics related to the services required by their duties. As stated in the Family Medicine Practice Regulation [10], these midwives are obliged:

**Figure 1:**
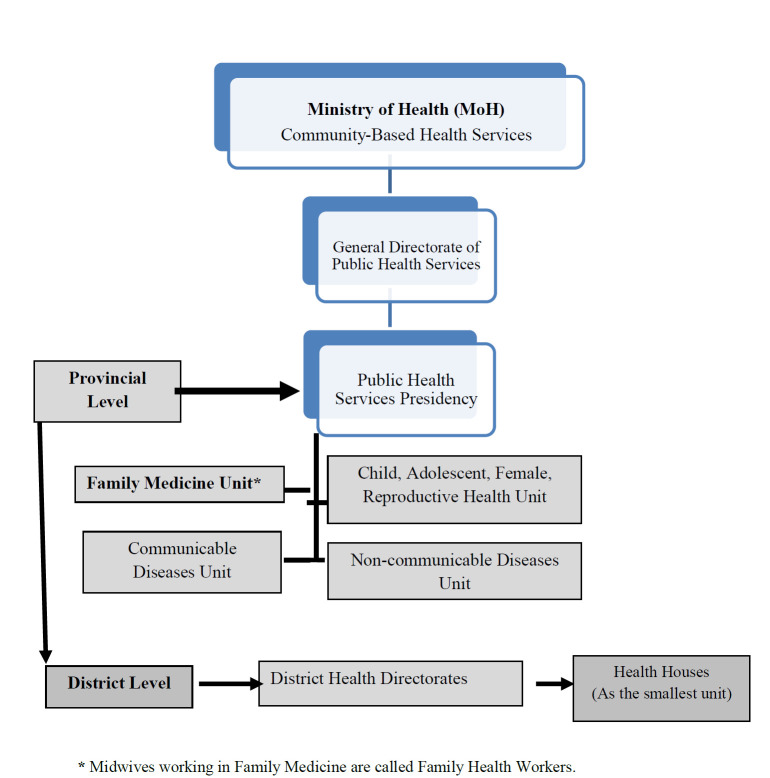
Community-based health services.

- to provide preventive, therapeutic, and rehabilitative health services,
- to keep health records and statistics related to the services required by their duty,
- to carry out preventive health services such as personal immunization,
- to perform cancer screening regarding individuals’ age, sex, and disease groups,
- to monitor individuals in terms of chronic diseases, pregnancy, puerperality,
- to monitor newborns’, infants’, children’s, adolescents’, adults’, and older adults’ health,
- to measure and record the vital signs of individuals who present to the centre,
- to administer drugs under the supervision of the physician,
- to provide wound care, to have materials and devices ready for service,
- to take samples for necessary examinations and send the samples taken to the relevant laboratory, to participate in in-service training determined by the institution.

## MIDWIVES IN THE COVID-19 SURVEILLANCE SYSTEM

Since the first COVID-19 cases in Turkey, several interventions have been conducted to prevent and control the pandemic. Before deciding on a method, its basic characteristics and the country’s condition should be considered [4].

**Figure 2:**
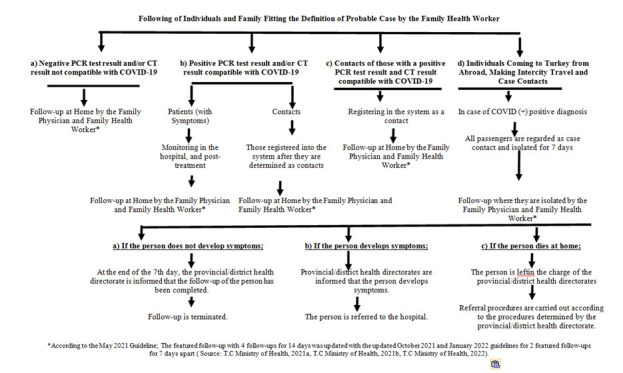
Following of individuals and family fitting the definition of probable case by the family health worker.

People diagnosed with the disease in Turkey are tracked by the Public Health Management System (PHMS), which immediately registers patients who are tested positive. There are generally two outcomes after the patients are tracked and registered in the PHMS: they are either followed-up at home or followed-up and treated in the hospital. Negative cases, contacts of patients with COVID-19, those entering the province from other cities, those in the ≥65-year-old age group, and certain positive cases are followed up by home isolation committees. Individuals thought to be at risk for infection and contagiousness and those meeting the possible case definition should be followed up by the FHC; the subsequent steps after follow-up are shown in **Figure 2** [11].

Restructuring routine and COVID-19 follow-ups of pregnant women, infants, children, chronically ill people, adults, and older adults presenting to the FHC is crucial in preventing them from negatively affecting one another and fully integrating. To avoid disruption, follow-ups and applications have been rearranged so that the groups under surveillance do not constitute a source of infection, that COVID-19 infection is not transmitted from the institution, and that health care workers including midwives do not constitute a source of infection or that they are not infected. The following steps are taken to achieve this;

- To prevent transmission, children are contacted by phone to ensure they do not stay in the same waiting area as patients presenting to the centre. Other individuals who need to be followed up are also given an appointment by phone.
- Patients are taken into the centre and given health services individually in a private room.
- After each intervention, rooms are cleaned to decrease the risk of infection. Patients with COVID-19 complaints are examined separately in a ventilated examination room that provides the necessary physical conditions to prevent transmission.
- A triage area has been created where patients are triaged appropriately.
- Upon continuation of immunization, pregnant women and parents of newborns who are invited for vaccination or follow-up and are worried about contamination and do not want to bring their babies to the centre are informed about the safety precautions taken in the family health centre.
- Individuals who do not want to come to the FHC due to the possibility of infection are visited at home to complete the necessary follow-ups and neonatal screening program, and informed that vaccination can only be performed in the FHC.
- Midwives provide counselling during the follow-ups and refer individuals with symptoms to relevant health institutions.
- Family physicians and midwives working in the FHC follow the contacts of quarantined patients with COVID-19 and follow-up patients with negative tests and their contacts (**Figure 2**)
- Midwives working in FHC also play an active role in enabling disadvantaged groups in society such as the disabled, because they are vulnerable to and are seriously affected by the COVID-19 pandemic.
- Midwives provide counselling for individuals, families, and communities and share guidelines prepared by the MoH with relevant people and act in accordance with the guidelines
- Within the scope of the vaccination program, midwives working in FHC also perform vaccination services for the population they serve.

During the pandemic, health services such as immunization, baby/child follow-up, and pregnancy follow-up have continued without any interruption and without unequal treatment. To maintain the services in the best conditions, the primary duty of the midwives working in the FHC is to protect their own health and that of the person(s) they serve.

## CONCLUSION

In Turkey, as in the rest of the world, community-based services have been negatively affected by the COVID-19 pandemic. Despite evidence suggesting otherwise, community services have been restricted and treatment services have been prioritized during the pandemic [3]. Community-based midwifery services have been more than other community service providers and were forced to provide most care online. Restrictions were introduced in important services such as pregnancy follow-up, baby care, and breastfeeding support. Due to the pandemic, families and pregnant women did not want or could not come to the family health centres. They were reached by telephone and followed up. In this case, counselling or information was given according to the patients’ self-reported situations, as midwives could not directly examine or observe them. This posed the risk of decreasing quality individual and family-centred maternal and infant health care.

Midwives working in every branch of primary health care have taken an active role in both pandemic management and health protection and maintenance by participating in preventive health services since the COVID-19 pandemic emerged:

- Midwives not only have to care for pregnant women, newborns, infants, women aged 15-49, and the elderly population who are in the at-risk groups in FHC, but also protect themselves and their families from infection. Midwives who are conscious of their responsibilities as health workers have continued to carry out their basic tasks despite pandemic-related interruptions. They are faced with both emotional and work overload. Countries worldwide should ensure systems and processes aimed at supporting and providing care for midwives and all other health professionals throughout the pandemic.
- Appropriate strategies should be developed and implemented to reduce the risk of post-traumatic stress disorder and burnout. Psychological support, social commitment, and care are essential for health workers.
